# Manganese-enhanced MRI detects live human amnion-derived mesenchymal stem cells in vivo after transplantation and restoration of myocardial function in a pig ischemia-reperfusion injury model

**DOI:** 10.1186/1532-429X-14-S1-P62

**Published:** 2012-02-01

**Authors:** Rajesh Dash, Ildiko Toma, Fumiaki Ikeno, Jennifer K Lyons, Shahriar Heidary, Marie-Claude Parent, INing E Wang, Xiaohu Ge, Jaehoon Chung, Justin Lam, Paul J Kim, Kaori Nakagawa, Svetlana Lyalina, Grace Do, Robert C Robbins, Michael V McConnell, Alan Yeung, Phillip Harnish, Phillip C Yang

**Affiliations:** 1 Cardiovascular Medicine, Stanford University, Stanford, CA, USA; 2 Cardiology, University of Illinois-Chicago, Chicago, IL, USA; 3 Cardiac Surgery, Stanford University, Stanford, CA, USA; 4 Electrical Engineering, Stanford University, Stanford, CA, USA; 5 Eagle Vision Pharmaceuticals Corp., Downington, PA, USA

## Summary

Human Amnion-derived Mesenchymal Stem Cells (hAMSCs) were transplanted into the infarct and peri-infarct regions of a pig ischemia-reperfusion model. The hAMSC therapy improved cardiac systolic function post-MI, compared to control animals, and Cardiac MRI with Manganese-Enhanced MRI (MEMRI) was able to detect increased CNR from live populations of hAMSCs within infarct and peri-infarct zones, as confirmed by human nuclear antigen (hNA) immunostaining.

## Background

Stem cell-mediated restoration of cardiac function after myocardial infarction (MI) has been reported; however, it remains unclear whether transplanted stem cells survive and engraft in the heart following transplantation. To investigate stem cell viability in vivo, our laboratory previously validated a Manganese-Enhanced MRI (MEMRI) contrast agent, EVP-1001-1 (Eagle Vision Pharmaceuticals Corp.), that specifically enters live cardiac tissue in a pig ischemia-reperfusion (IR) injury model. T1-weighted MRI imaging following EVP-1001 injection delineates infarct from remote and border zones. EVP-1001-1 is also taken up strongly by live stem cells. In this study, EVP-1001-1 was used to track human amnion-derived mesenchymal stem cells (hAMSCs) after transplantation into pig hearts post-IR.

## Methods

Five adult farm pigs underwent one-hour IR to the mid LAD coronary artery. One week post-IR, pigs hearts were injected with either hAMSCs (total 50 million cells per heart, n=3) or normal saline (NS, n=2) into ~8 peri-infarct and infarct zones, using fluoroscopic guidance and a BioCardia catheter injection system (Biocardia, Inc.). Cardiac MRI was performed to assess ventricular function (ejection fraction, EF%), infarct % by Delayed Gadolinium Enhancement MRI (DEMRI), and evidence for cell survival using MEMRI at serial timepoints post-IR.

## Results

**Figure 1:**
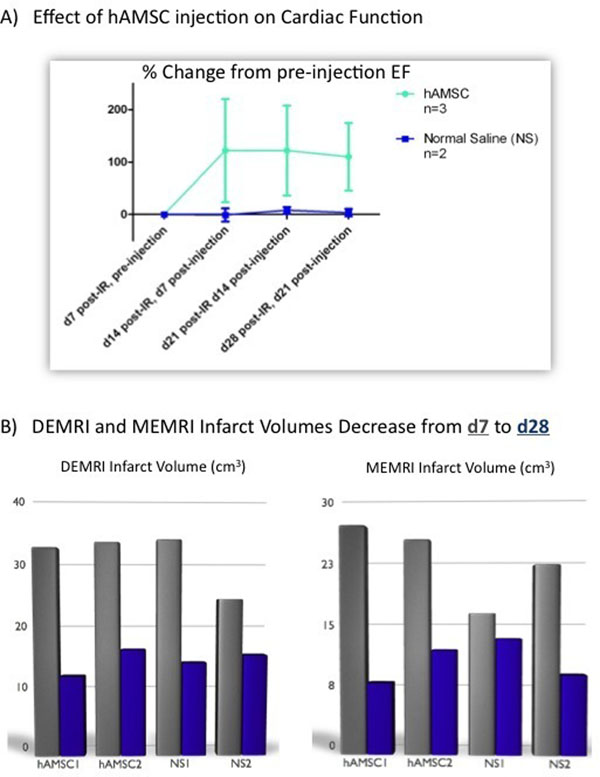
A) Effect of hAMSC delivery on EF. hAMSC-treated hearts exhibit increased EF versus NS control hearts (p>0.05), indicating a functional restoration post-IR injury; B) DEMRI and MEMRI infarct volumes reduce over time from d7 to d28 post-IR. Although DEMRI volume reductions are similar between hAMSC and NS hearts, MEMRI defect reduction tends to be greater in hAMSC hearts.

**Figure 2:**
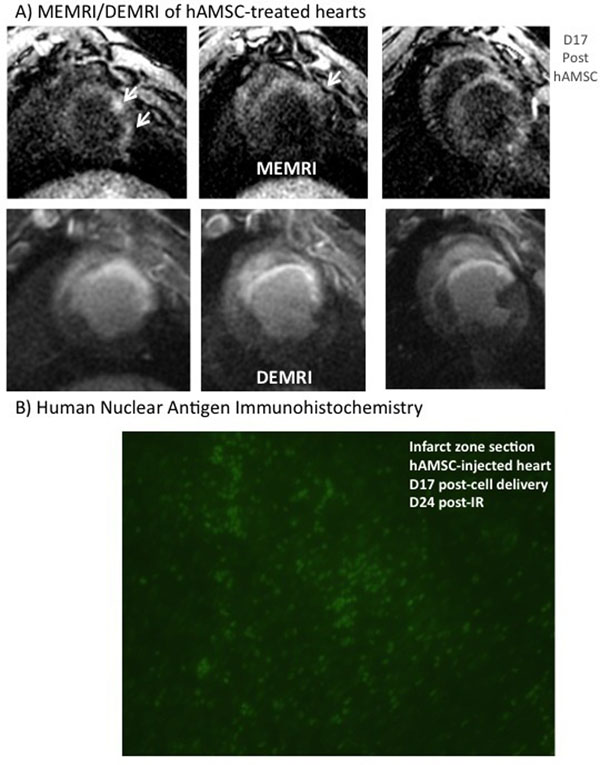
A) MEMRI Signal in Infarct Zone. Note the discrete regions of increased signal intensity on MEMRI imaging (top panels, white arrows on consecutive shot-axis images) within areas of infarction, as denoted by corresponding DEMRI panels below. B) Immunohistochemistry for human Nuclear Antigen (hNA) taken from the infarct zone of a hAMSC-treated heart. Bright green cells represent positive-staining hAMSCs that are abundant within the infarct zone 17 days after transplantation, representing a potential source for increased MEMRI signal in this region.

The average EF was similar at baseline (57±4% (n=5)) and 1 week post-IR (pre-injection EF: 24±6%). DEMRI infarct size was also similar between the groups (Figure 1B). hAMSC swine exhibited higher EFs at 1- and 2-weeks post-hAMSC delivery (d14 and d21 post-IR), compared to NS-injected animals (Figure 1). Both DEMRI and MEMRI infarct volumes decreased from day 7 to day 21 post-IR in both groups (Figure 1B). MEMRI defect volume decreased to a higher degree in hAMSC hearts. MEMRI also revealed discrete regions of high contrast-to-noise ratio (CNR) within infarct and peri-infarct zones in hAMSC-treated animals (hAMSC CNR: 8.6±1.4*; NS CNR: 4.9±0.8, n=3, *p<0.05), suggesting increased Manganese uptake by live stem cell populations within the infarct zone (Figure 2A). Human nuclear antigen (hNA) immunostaining of pig heart sections confirmed intact populations of transplanted hAMSCs within the infarct zones up to 17 days post-transplantation (Figure 2B).

## Conclusions

hAMSC delivery to the peri-infarct and infarct zones post-IR improves left ventricular systolic function compared to saline-injected or control animals. Increased MEMRI CNR of the infarct zone is associated with positive hNA staining in hAMSC-treated hearts, providing evidence for live hAMSC populations weeks after cell delivery that may be contribute to improved ventricular function. MEMRI of transplanted hAMSCs may allow longitudinal tracking of transplanted cells in vivo.

## Funding

NIH-NHLBI: K08 (RD), R01 (PY).

